# Risk factors/cofactors for heightened anaphylaxis severity in Japanese adults: A 10-year single-center retrospective cohort study

**DOI:** 10.1016/j.waojou.2025.101062

**Published:** 2025-05-08

**Authors:** Makoto Nojo, Shintaro Suzuki, Tomoki Uno, Yoshito Miyata, Tanaka Akihiko, Hironori Sagara

**Affiliations:** a Division of Respiratory Medicine and Allergology, Department of Internal Medicine, Showa University School of Medicine, Showa University Hospital, 1-5-8, Shinagawa-Ku, Tokyo 142-8666, Japan; b Department of Medical Education, Showa University School of Medicine, Showa University Hospital, 1-5-8, Shinagawa-Ku, Tokyo 142-8666, Japan

**Keywords:** Adult, Anaphylaxis, Augmentation factor, Disease severity, Smoking

## Abstract

**Background:**

Anaphylaxis is a severe and life-threatening allergic reaction. Although the risk factors/cofactors for anaphylaxis vary between countries and regions, limited information is available on these factors within the Japanese context. Therefore, we aimed to discern risk factors/cofactors associated with heightened anaphylaxis severity in Japanese adults.

**Methods:**

In total, 507 adult patients with anaphylaxis who visited our clinic (Tokyo, Japan) between January 2010 and June 2020 were included in the analysis. Data on patient backgrounds, clinical characteristics, and causative allergens were extracted from patients’ medical records. We retrospectively analyzed information on patient background and clinical characteristics associated with an increased severity of anaphylaxis. Logistic regression modeling was used to identify background features and clinical characteristics that contribute to anaphylaxis severity.

**Results:**

Multivariate analysis revealed that age, smoking history, asthma, and alcohol consumption at the onset of anaphylaxis were significant risk factors contributing to the increased severity of anaphylaxis. Moreover, drug-induced anaphylaxis was associated with heightened severity than food, anisakis and other allergens.

**Conclusion:**

We successfully identified risk factors/cofactors contributing to the heightened severity of anaphylaxis among adults in Japan. Additionally, our findings suggest that alcohol consumption and smoking are related to anaphylaxis severity in adults. The insights derived from this study will assist in identifying more effective preventive measures and treatment strategies in clinical practice.

## Introduction

Anaphylactic shock is a severe and acute allergic reaction that can prove fatal. Although anaphylaxis cases in adults were previously thought rare, their incidence is gradually increasing.^1^ The number of adult anaphylaxis cases is not significantly different from the number of pediatric anaphylaxis cases.^2^ Identifying and eliminating the trigger (particularly in cases of food or drug allergy) is crucial to prevent the onset of anaphylaxis, given the absence of medications for treating and managing the underlying condition. Three factors predominantly influence the severity of anaphylaxis: age/life stage, comorbidities/medication history, and anaphylaxis-augmenting factors.^3^ According to the World Allergy Organization (WAO) guidance, age/life stage is a particularly important factor, with infants, adolescents, young adults, pregnant women, and older individuals exhibiting heightened severity of anaphylaxis.^3^ Previous multicenter epidemiological studies in Europe and research on adult patients with anaphylaxis have indicated that anaphylaxis severity increases with age and life stage.^4^^,^^5^ Moreover, triggers, comorbidities, medications, and physical conditions may influence anaphylaxis severity differently in adults compared with that in children and individuals of other life stages, thereby affecting prognosis. Consequently, alterations in anaphylaxis severity and status may affect prognosis differently between adults and children.

Factors that exacerbate anaphylaxis (referred to as cofactors or augmentation factors) include male sex; exercise; alcohol consumption; psychological stress; use of oral non-steroidal anti-inflammatory drugs (NSAIDs), β-receptor blockers, and angiotensin-converting enzyme inhibitors/angiotensin II receptor blockers (ACE-I/ARBs); and underlying conditions, such as mastocytosis, asthma, atopic dermatitis, and cardiovascular disease (CVD).^4^ A multicenter European registry study revealed cofactor involvement in approximately 30% of adult anaphylaxis cases caused by food allergies,^6^ which increased to 40% in Japanese adult anaphylaxis cases.^7^ Considering the importance of these cofactors, avoidance of the cofactors related to suspected allergen ingestion can reduce the severity of anaphylaxis,^8^ highlighting the importance of identifying cofactors in adult anaphylaxis cases. As anaphylaxis in pediatric patients are primarily attributed to common food allergies with minimal cofactor involvement, a few studies have focused on cofactors such as the history of alcohol consumption, smoking, and adult-specific comorbidities that are unique to adulthood.

In this study, we aimed to identify risk factors/cofactors contributing to anaphylaxis severity in adults and compared the characteristics of adult anaphylaxis cases across different life stages.

## Methods

### Participant selection

In this study, we enrolled 531 adult patients who visited the respiratory medicine and allergology outpatient clinic of our university hospital and were diagnosed with anaphylaxis between January 2010 and June 2020. Subsequently, a specialized search was conducted to identify anaphylaxis-causing antigens. Subsequently, 24 individuals were excluded owing to incomplete data or their withdrawal from the study; finally, the remaining 507 patients were included in the study. We categorized the participants into 3 groups for comparison: the youth group (20–39 years), the middle-aged group (40–64 years), and the older age group (65 years or older). Our facility is located in the urban center of Tokyo, and in 2020, there were 220 new patients who visited our facility for the purpose of identifying the cause of allergies.

### Extracting information from medical records

We examined symptoms, severity, causative antigen, and treatment at the time of anaphylaxis diagnosis from medical records, as well as background features and clinical characteristics that contributed to anaphylaxis severity. These factors, outlined in the Japanese Anaphylaxis Guidelines derived from the WAO Anaphylaxis Guidance, were considered risk factors/cofactors that could exacerbate anaphylaxis severity. For statistical analysis, parameters such as exercise, alcohol consumption, coexisting allergic diseases (including bronchial asthma), coexisting CVD, coexisting psychiatric disorders, medical history (ACE-I/ARBs, β-receptor blockers, sedatives, antidepressants), and general social history (drinking and smoking history) were considered.

### Assessing and classifying anaphylaxis severity

The severity of anaphylaxis remains difficult to quantify, as physicians primarily evaluate the presence of qualitative symptoms. In this study, anaphylaxis severity was determined based on the presence of induced symptoms (Table 1), according to the grading in the Japanese Anaphylaxis Guidelines,^9^ which were modified from the symptom grading for anaphylactic reactions in the EAACI guidelines.^10^ Objective symptoms (including a range of skin, respiratory, and gastrointestinal symptoms [vomiting and diarrhea]) were considered positive criteria. Symptoms were discussed and graded by 2 allergologists certified by the Japanese Society of Allergology; severe anaphylaxis was classified as grade III and moderate anaphylaxis as grade II.

**Table 1:** Symptom grading.

Symptom grading			
	1 (Mild)	2 (Moderate)	3 (Severe)
**Skin**	Localized urticaria, exanthema, wheal, pruritus	Generalized urticaria, exanthema, wheal, pruritus	–
	Swollen eyelid or lip	Swollen face	–
**Gastrointestinal tract**	Pruritus of the throat or oral cavity	Throat pain	–
	Mild abdominal pain	Moderate abdominal pain	Cramps
	Nausea, emesis, diarrhea	Recurrent emesis, diarrhea	Continuous emesis, loss of bowel control
**Respiratory tract**	Intermittent cough, nasal congestion, sneezing, rhinorrhea	Repetitive cough	Persistent cough, hoarseness, “barking” cough
	–	Chest tightness, wheezing detectable via auscultation	Audible wheezing, dyspnea, cyanosis, saturation <92%, swallowing or speaking difficulties, throat tightness, respiratory arrest
**Cardiovascular**	–	Pale face, mild hypotension, tachycardia (increase by >15 beats/min)	Hypotension, dysrhythmia, severe bradycardia, cardiac arrest
**Neurological**	Change in activity level, tiredness	Light-headedness, feeling of “pending doom,” somnolence, headache	Confusion, loss of consciousness, incontinence

The severity score was based on the organ system that was most affected by the symptoms.^9^ Hypotension was defined as a systolic blood pressure of <70 mm Hg (age: 1 month to 1 year), <70 mm Hg + (2 × age) (age: 1–10 years), and <90 mm Hg (age: >11 years). Mild hypotension was defined as a systolic blood pressure of <80 mm Hg (age: 1 month to 1 year), <80 mm Hg + (2 × age) (age: 1–10 years), and <100 mm Hg (age: >11 years). Wheezing detectable via auscultation was defined as mild wheezing that was audible only through a stethoscope. Audible wheezing was defined as wheezing audible without a stethoscope. This definition was modified using the anaphylactic symptom grading of the EAACI guidelines.^10^

### Classifying causative antigens

Causative allergens were broadly categorized as (1) food-related (causing food-dependent, exercise-induced anaphylaxis; oral mite allergy; and pollen food allergy syndrome); (2) *Anisakis*; (3) drugs; and (4) others (items not classified into these 3 groups, such as insect stings, natural dyes, idiopathic causes, and unknown causes). The anaphylaxis-causing antigen was diagnosed by an allergist certified by the Japanese Society of Allergology, adhering to diagnostic criteria specified by medical societies, and the Japanese Society of Allergology and the Japanese Society of Pediatric Allergy. In Japan, allergy to *Anisakis* (a parasite that contaminates seafood) is a common trigger for acute allergic reactions in Japanese adults who consume raw seafood.

As *Anisakis* allergy is challenging to diagnose through skin or challenge testing and a definitive diagnostic method has not been established owing to the low specificity of *Anisakis* allergy-specific immunoglobulin E (IgE) testing, the following diagnostic approaches were employed: 1) assessing the history of seafood ingestion, including cooked seafood, within 24 h, 2) determining *Anisakis* allergy-specific IgE elevation (specific IgE level >0.35 Ua/mL), 3) exclusion of other allergens as an essential criterion, and 4) considering a history of suspected *Anisakis* allergy as adjuncts to the clinical diagnosis.^11^^,^^12^

In Japan, mastocytosis is very rare, and there were no cases in this study either. For this reason, we did not list it as a risk factor or causative antigen.^13^

### Statistical analysis

Numerical data are presented as mean ± standard deviation. The Fisher's test of variance was used to compare values among multiple groups; statistical significance was set at p < 0.05. Logistic regression modeling was used to identify background features and clinical characteristics that contribute to anaphylaxis severity. To maintain the assumptions of logistic regression, reactions reported more than once by the same participants were excluded. Thus, only the first reported anaphylactic event was included in the final database for patients with multiple reactions. Multivariate analysis was performed using age, sex, comorbidities (rhinitis, asthma, atopic dermatitis, cardiac disease, and psychiatric disease), concomitant medications (anxiolytics and sleeping pills), reaction elicitors (limited to food, *Anisakis*, drugs, and other), and lifestyle (drinking history, smoking history, drinking history immediately prior to anaphylaxis, exercise history immediately prior to anaphylaxis). Moreover, patients on beta-blocker treatment were excluded from the multivariate analysis owing to their limited representation in the dataset (10 [2.0%] participants on beta-blockers and 4 [0.8%] on ACE-I/ARBs) and their inclusion could compromise the statistical stability of the results. In the multivariate analysis, the odds ratio (OR) for all pairwise comparisons was obtained from the same logistic model. To illustrate the relationship between age and severity score, we generated a scatter plot with a smoothing spline and confidence interval (CI). The statistical analyses were performed using JMP ver. 17 (SAS Institute, Cary, NC, USA).

## Results

### Characteristics by age group

The mean age of the 507 eligible patients was 44.2 ± 16.3 years, with 201 (39.6%) patients being men. The distribution included 230 (45.4%) patients in the youth group, 207 (40.8%) in the middle-aged group, and 70 (13.8%) in the older age group (Table 2).

**Table 2:** Classification of characteristics by age.

Characteristic	All % (n = 507)	Youth % (n = 230)	Middle-aged % (n = 207)	Older age % (n = 70)
**Anaphylaxis severity**	64.9 (329)	54.8 (126)	70.5 (146)	81.4 (57)
**Male sex**	39.7 (201)	37.0 (85)	42.5 (88)	40 (28)
**History of allergy**	67.3 (341)	73.9 (170)	62.3 (129)	60.0 (42)
Bronchial asthma	27.2 (138)	30.4 (70)	24.2 (50)	25.7 (18)
Atopic dermatitis	13.6 (69)	21.7 (50)	7.3 (15)	5.7 (4)
Allergic rhinitis	54.1 (274)	58.3 (134)	50.1 (104)	51.4 (36)
**History of present illness**				
CVD	15.6 (79)	1.7 (4)	19.3 (40)	50.0 (35)
Psychiatric disorders	8.1 (41)	2.2 (5)	9.2 (19)	24.3 (17)
**Medical history**				
β-blockers	2.0 (10)	0.0 (0)	2.9 (6)	5.7 (4)
ACE-I/ARBs	0.8 (4)	0.0 (0)	0.5 (1)	4.3 (3)
Antidepressants	3.0 (15)	1.3 (3)	4.4 (9)	4.3 (3)
Sedatives	4.9 (25)	0.43 (1)	3.9 (8)	22.9 (16)
**Social history**				
Smoking history	29.6 (150)	20.0 (46)	40.1 (83)	30.0 (21)
Alcohol history	51.9 (263)	46.1 (106)	59.9 (124)	47.1 (33)
**Other cofactors**				
Alcohol in anaphylaxis	23.3 (118)	16.1 (37)	30.9 (64)	24.3 (17)
Exercise in anaphylaxis	10.8 (55)	10.4 (24)	12.6 (26)	7.1 (5)
**Causative allergens**				
Food	47.14 (249)	54.35 (125)	41.55 (86)	40.00 (28)
*Anisakis*	23.27 (118)	15.22 (35)	28.99 (60)	32.86 (23)
Drug	10.26 (52)	8.26 (19)	10.63 (22)	15.71 (11)
Other	19.33 (98)	22.17 (51)	18.84 (39)	11.43 (8)

ACE-I/ARB, angiotensin-converting enzyme inhibitors/angiotensin II receptor blocker; CVD, cardiovascular disease.

### History of allergy

Among all patients, 27.2% had bronchial asthma, 13.6% had atopic dermatitis, and 55.4% had allergic rhinitis (Table 2). A higher prevalence of allergies (73.9%) was observed in the youth group (especially atopic dermatitis and asthma) than in the middle-aged (62.3%) and older age (60.0%) groups.

### History of present illness and medical history other than allergy

Among all patients, 15.6% had CVD, 8.1% had psychiatric disorders, and 2.0%, 0.8%, 3.0%, and 4.9% were on beta-blockers, ACE-I/ARBs, antidepressants, and sedatives, respectively (Table 2). The older age group had a higher incidence of underlying medical conditions, including CVD and psychiatric disorders than the younger and middle-aged groups. Notably, none of the patients in the youth group were on beta-blockers or ACE-I/ARBs; however, the usage of these medications increased with age. Additionally, a higher proportion of patients in the older age group used sleep aids than those in the youth and middle-aged groups.

### Social history and other cofactors

Among all patients, 29.6% had a history of smoking and 51.9% had a history of alcohol consumption (Table 2). Furthermore, 23.3% of patients had been consuming alcohol and 10.8% had been engaging in exercise immediately before anaphylaxis. The patients in the middle-aged group had a higher prevalence of social history elements, such as history of smoking, alcohol consumption, and exercise at the onset of anaphylaxis than those in the other 2 groups.

### Anaphylaxis triggers

Food was the predominant causative antigen (239 cases, 47.1%), followed by *Anisakis* allergy (118 cases, 23.3%), other causes (98 cases, 19.3%), and drugs (52 cases, 10.2%) (Table 3). Although food remained the primary cause across all life stages, its predominance decreased with advancing age, inversely correlating with increased rates of *Anisakis* allergy and drug-related triggers. The *Anisakis* allergy rate in the older age group was approximately twice that in the youth group. Food allergies were recorded in 239 cases and included diverse triggers, with wheat, crustaceans, fruits, vegetables, and soybeans being the most common, in the given order. Most foods (except for vegetables, tree nuts, and animal meat) were more commonly consumed by individuals in the younger, middle-aged, and older age groups. Further details are provided in the accompanying table (Table 2). Drug allergies were present in 52 cases, with allergies of antibacterial agents, nonsteroidal anti-inflammatory drugs (NSAIDs), cold remedies, local anesthetics, and antispasmodics ranking the highest. Of the remaining 98 cases, 82 had allergies of unknown origin (unknown), including 8 cases of stings from mites and bees.

**Table 3:** Causative allergens of anaphylaxis by age.

Causative allergen	All % (n = 507)	Youth % (n = 230)	Middle-aged % (n = 207)	Older age % (n = 70)
**Foods**	47.14 (239)	54.35 (125)	41.55 (86)	40.00 (28)
Wheat	11.83 (60)	11.30 (26)	14.01 (29)	7.14 (5)
Crustaceans	9.66 (49)	13.48 (31)	6.76 (14)	5.71 (4)
Fruits	5.72 (29)	5.65 (13)	4.35 (9)	10.00 (7)
Vegetables	3.16 (16)	1.74 (4)	4.35 (9)	4.29 (3)
Soy	2.17 (11)	1.74 (4)	1.93 (4)	4.29 (3)
Tree nuts	2.17 (11)	3.04 (7)	1.93 (4)	0 (0)
Buckwheat	1.78 (9)	2.17 (5)	1.45 (3)	1.43 (1)
Egg	1.58 (8)	2.17 (5)	0.48 (1)	2.86 (2)
Oral mite allergy	1.38 (7)	1.74 (4)	1.45 (3)	0 (0)
Peanuts	1.38 (7)	1.74 (4)	0.48 (1)	2.86 (2)
Shellfish	1.38 (7)	2.17 (5)	0.97 (2)	0 (0)
Fish egg	0.99 (5)	1.74 (4)	0.48 (1)	0 (0)
Milk/Dairy	0.79 (4)	1.30 (3)	0 (0)	1.43 (1)
Fish	0.59 (3)	0.87 (2)	0.48 (1)	0 (0)
Meat	0.39 (2)	0 (0)	0.97 (2)	0 (0)
Mollusk	0.20 (1)	0 (0)	0.48 (1)	0 (0)
Other foods	1.97 (10)	3.48 (8)	0.97 (2)	0 (0)
***Anisakis***	23.27 (118)	15.22 (35)	28.99 (60)	32.86 (23)
**Drugs**	10.26 (52)	8.26 (19)	10.63 (22)	15.71 (11)
Antibiotics	3.16 (16)	3.04 (7)	2.90 (6)	4.29 (3)
Quinolone	1.78 (9)	3.04 (7)	0.97 (2)	0 (0)
Cephalosporin	0.79 (4)	0 (0)	1.45 (3)	1.43 (1)
Penicillin	0.39 (2)	0 (0)	0 (0)	2.86 (2)
Minocycline	0.20 (1)	0 (0)	0.48 (1)	0 (0)
NSAIDs	2.56 (13)	3.04 (7)	1.45 (3)	4.29 (3)
Propionic acids	0.99 (5)	1.74 (4)	0.48 (1)	0 (0)
Acetic acids	0.39 (2)	0 (0)	0.48 (1)	1.43 (1)
Unknown	1.18 (6)	1.30 (3)	0.48 (1)	2.86 (2)
Cold medicine	1.38 (7)	0.87 (3)	1.45 (3)	1.43 (1)
Local anesthetics	0.59 (3)	0 (0)	0.97 (2)	1.43 (1)
Antispasmodics	0.59 (3)	0 (0)	1.45 (3)	0 (0)
Contrast media	0.39 (2)	0 (0)	0.97 (2)	0 (0)
Other drugs	1.58 (8)	1.30 (3)	0.97 (2)	4.29 (3)
**Other**	19.33 (98)	22.17 (51)	18.84 (39)	11.43 (8)
Unknown	16.17 (82)	18.70 (43)	15.46 (32)	10.00 (7)
Bee sting	1.58 (8)	1.30 (3)	1.93 (4)	1.43 (1)

Food was the most prevalent allergen (47.14%), followed by *Anisakis* (23.27%), drugs (10.26%), and others (19.33%). While food was the primary allergen across all life stages, its predominance diminished in the middle-aged and older age groups, contrasting with increasing rates of *Anisakis* and drug allergies.

NSAID, non-steroidal anti-inflammatory drug.

### Age significantly influences anaphylaxis severity

**Fig. 1:**
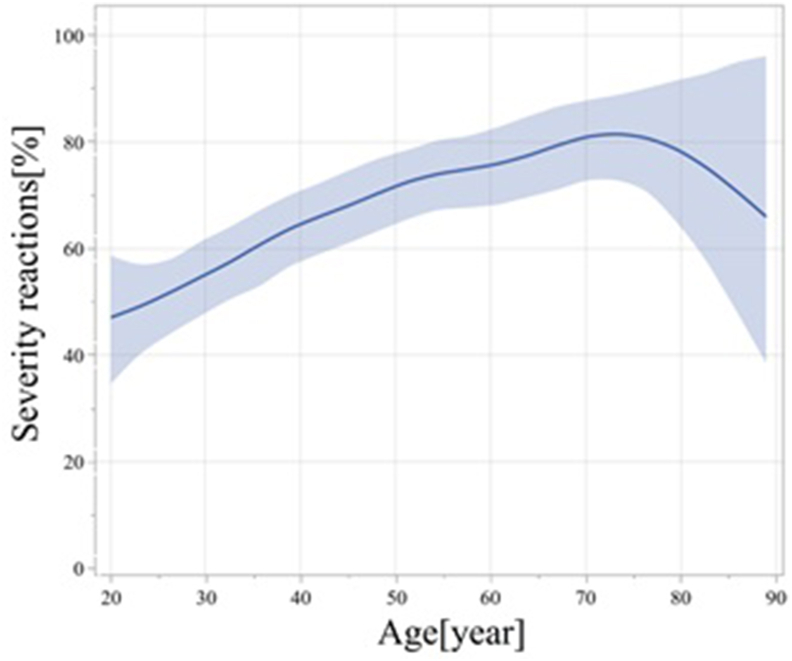
Age significantly influences anaphylaxis severity. Solid curves: fitted Loess curves with 95% confidence intervals in gray. The odds ratio for age demonstrated a 20.8% increase (95% CI: 3.89%–40.6%) in severe anaphylaxis for every 10-year age increment. Although the very older age group exhibited a non-linear relationship, severity increased with age, especially in the older age group.

Among all patients, 178 (35.1%) and 329 (64.9%) individuals were classified under Grade 2 and 3, respectively (Table 2). In terms of severity by age, 54.8% of the youth group, 70.5% of the middle-aged group, and 81.4% of the older age group experienced severe anaphylaxis, suggesting that the severity of the disease increases as individuals get older. Although age extremes did not exhibit a linear relationship, anaphylaxis severity increased with age, with significantly higher severity observed in the older age group (Fig. 1). We observed that every 10-year increase in age was associated with a 20.8% (95% CI: 3.89%–40.6%) increase in the OR for experiencing a severe anaphylactic event, assuming that all other variables were equal. The OR for age was 3.70 (95% CI: 1.30–10.50) (Table 4).

**Table 4:** Multivariate analysis of anaphylaxis severity.

Characteristic	Odds ratio	95% CI	p value
**Age**^a^	1.21	1.04–1.41	0.014
**Sex: Male**	0.89	0.57–1.39	0.61
**History of allergy**			
Bronchial asthma	1.82	1.13–2.91	0.013
Atopic dermatitis	0.95	0.53–1.73	0.88
Allergic rhinitis	1.05	0.70–1.58	0.80
**Present illness**			
CVD	1.60	0.78–3.29	0.20
Psychiatric disorders	0.76	0.15–3.90	0.75
**Drug history**			
Antidepressants	1.45	0.24–8.57	0.68
Sedatives	1.04	0.18–6.10	0.96
**Social history**			
Smoking history	1.83	1.10–3.04	0.02
Alcohol history	0.80	0.50–1.27	0.34
**Other cofactors**			
Alcohol in anaphylaxis	2.65	1.44–4.88	0.0018
Exercise during anaphylaxis	4.37	1.95–9.82	0.0003
**Allergen**			
Drug vs. Other	3.14	1.38–7.10	0.0062
Drug vs. Food	2.94	1.37–6.30	0.0056
Drug vs. *Anisakis*	1.71	0.74–3.93	0.21
*Anisakis* vs. Other	1.83	0.98–3.45	0.060
*Anisakis* vs. Food	1.72	0.98–3.02	0.058
Food vs. Other	1.07	0.62–1.83	0.82

Except for the causative allergens and ages, the most contributing factors to the severity of anaphylaxis were found to be exercise in anaphylaxis, alcohol in anaphylaxis, smoking, and bronchial asthma, in that order.

CVD, cardiovascular disease.

a Odds ratios for age are presented in increments of 10 years.

### Smoking history as a contributor to anaphylaxis severity

The multivariate analysis revealed that smoking history correlated with anaphylaxis severity (OR: 1.83, 95% CI: 1.10%–3.04%, p = 0.02; Table 4). A similar multivariate analysis conducted on 150 smokers revealed that bronchial asthma was the primary factor associated with anaphylaxis severity in smokers (OR: 8.39, 95% CI: 2.00–35.19, p = 0.02), followed by CVD (OR: 8.79, 95% CI: 1.62–47.8, p = 0.012) and causative antigen (drug). Conversely, factors contributing to anaphylaxis severity in non-smokers included exercise at the time of anaphylaxis, age, and alcohol consumption at the time of anaphylaxis.

### Alcohol consumption and exercise cofactors associate with anaphylaxis severity

The multivariate analysis revealed alcohol consumption during anaphylaxis and exercise as contributors to anaphylaxis severity (OR: 2.65, 95% CI: 1.44–4.88, p = 0.0018; and OR: 4.37, 95% CI: 1.95–9.82, p = 0.0003, respectively) (Table 4). In the participants, exercise was the factor most associated with exacerbated severity.

### Coexistence of bronchial asthma exacerbates anaphylaxis severity

The multivariate analysis of date of all 507 patients indicated an association between bronchial asthma and anaphylaxis severity (OR: 1.82, 95% CI: 1.13–2.91, p = 0.013) (Table 4). History of allergies other than asthma (allergic rhinitis and atopic dermatitis) was not associated with anaphylaxis severity (allergic rhinitis, OR: 1.05, 95% CI: 0.70–1.58, p = 0.80; atopic dermatitis, OR: 0.95, 95% CI: 0.53–1.73, p = 0.88). The multivariate analysis of date of 138 patients with bronchial asthma (similar to the above multivariate analysis) revealed that a history of smoking and alcohol consumption at the time of anaphylaxis were the crucial factors contributing to the severity of anaphylaxis. Furthermore, the factor contributing the most to the severity of anaphylaxis in patients with bronchial asthma was smoking history (OR: 8.17, 95% CI: 2.07–32.17, p = 0.0027).

### Causative allergens (particularly drugs) drive anaphylaxis severity

Among causative allergens, drugs were associated with increased anaphylaxis severity (Table 4). *Anisakis* was also associated with more severe anaphylaxis than other antigens; however, this difference was not statistically significant.

### Other comorbidities were not significantly associated with increased anaphylaxis severity

CVD was not significantly associated with anaphylaxis severity (OR: 1.60, 95% CI: 0.78–3.29, p = 0.20; Table 4). There was no significant association of anaphylaxis with psychiatric disorders, antidepressant use, and sedative use.

## Discussion

An important finding of this study is that cofactors only observed in adults (such as smoking history and alcohol consumption at the onset of anaphylaxis) influence anaphylaxis severity (Table 2, Table 4). Smoking history has not been reported to be associated with anaphylaxis, which has been the focus of several pediatric-based studies. In addition, our findings revealed that the coexistence of asthma, CVD, and antigen type were related to anaphylaxis severity, particularly in individuals with a history of smoking. This clarification enhances our understanding of adult patients who experience severe anaphylactic events.

In this study, we identified bronchial asthma, smoking history, involvement of alcohol consumption during anaphylaxis, involvement of exercise during anaphylaxis, and causative allergen as factors associated with the severity of anaphylaxis in adults (Table 4). The presence and contribution of risk factors/cofactors are peculiarities of adult anaphylaxis compared with that in children, where food allergy is a usual trigger and an important risk factor. Factors such as exercise, alcohol consumption, and medication use are considered crucial in the study of anaphylaxis severity and refractoriness in adults. Exercise and alcohol consumption at the time of anaphylaxis may exacerbate its severity by (1) increasing intestinal permeability, (2) increasing blood flow, (3) lowering the basophil/mast cell activation threshold, and (4) causing drowsiness from alcohol consumption and sedative uses, leading to missed initial pharyngeal symptoms, increased risk of accidental ingestion, and delayed initial response.^14^

We considered 2 possibilities through which smoking may play a role in anaphylaxis severity. First, smoking induces thymic stromal lymphopoietin (TSLP) expression and exacerbates allergic/type 2 inflammation. Second, smoking increases the severity of anaphylaxis owing to the increased severity of bronchial asthma.

Smoking promotes an immune response biased toward increased TSLP expression and Th2 inflammation and maintains high IgE levels and eosinophil counts in blood.^15^, ^16^, ^17^ Smoking-induced tracheal mucosal damage may exacerbate *trans*-airway sensitization to allergen exposure and enhance allergen exposure; in fact, specific IgE levels against aeroallergens, such as mites, cedar pollen, *Candida*, and crustaceans, are higher in smokers than in non-smokers.^17^, ^18^, ^19^ Therefore, smoking may exacerbate type 2 inflammation and increase *trans*-airway sensitization, which may increase the severity of anaphylaxis.

Second, smoking increases the severity of anaphylaxis owing to the increased severity of bronchial asthma. Although asthma is pointed out as a risk factor for fatal anaphylaxis in many studies and guidelines, recent findings suggest that asthma itself is not a strong predictor of more severe anaphylaxis, and that poor control of asthma increases the likelihood of patients developing severe anaphylaxis.^20^^,^^21^ Our results showed that the presence of bronchial asthma may contribute to the severity of anaphylaxis in smokers, and we speculate that increased severity of bronchial asthma owing to smoking influences the severity of anaphylaxis. Further studies considering the effect of the amount of smoking, route of allergy sensitization, severity of asthma, and presence of chronic obstructive pulmonary disease (COPD) as confounding factors are warranted since the present statistics did not confirm the route of allergy sensitization, the number of cigarettes smoked, whether the patient was a current or former smoker, the severity of bronchial asthma, or other confounding factors such as COPD.

In previous studies, age was a significant linear contributor to anaphylaxis severity up to 70 years of age. Age extremes did not exhibit a linear relationship, and anaphylaxis severity increased with age, with a significantly higher severity observed in the older age group. This finding may be related to the small number of patients older than 80 years, and a larger study addressing this gap is warranted. Anaphylaxis in older individuals is becoming more prevalent, highlighting the increasing importance of understanding and explaining the challenges posed by aging.^4^ In our previous study, the older population exhibited a high rate of cardiovascular and neurological symptoms, increased number of comorbidities and risk factors/cofactors, and a high rate of independently arriving at the emergency department without requesting emergency medical services despite the severity of the illness;^5^ these factors may contribute to the severity of anaphylaxis in older individuals.

As for comorbidities and medications contributing to the severity of anaphylaxis, “CVD” tended to insignificantly contribute to the severity of anaphylaxis. An additional sub-analysis of 3 CVD groups (hypertension-only, severe CVD [arrhythmia, heart failure, valvular disease, angina pectoris, and myocardial infarction], and non-CVD) revealed that patients in the severe CVD group may have more severe anaphylaxis than those in the non-CVD group (severe CVD group vs non-CVD, OR: 4.29, 95% CI: 0.87–21.10, p = 0.073). This partially correlates with the finding of a previous study indicating that CVD is likely to be involved in the severity of anaphylaxis.^4^

The multivariate analysis regarding the contribution of taking ACE-I/ARBs or β-receptor blockers to anaphylaxis severity could not be performed owing to the small number of patients. However, all patients taking ACE-I/ARB or beta-receptor blockers experienced severe anaphylaxis. This finding indicates that the use of ACE-I/ARBs or beta-blockers affects anaphylaxis severity. Moreover, psychiatric disorders, sedative use, and antidepressant use do not contribute to the severity of anaphylaxis. Thus, the tendency to induce drowsiness, miss the initial pharyngeal symptoms, increase the risk of accidental ingestion, and delay the initial response at the onset of anaphylaxis caused by the above history or medication may not contribute to anaphylaxis severity. The present statistical analyses included only a small number of patients with psychiatric disorders or those taking antidepressants. Furthermore, psychiatric disorders included insomnia, panic disorder, depression, and other disorders, which may have led to inadequate results. Therefore, additional large-scale surveys are warranted.

Among the causative allergens, drugs contributed to anaphylaxis severity, as previously reported;^4^ moreover, *Anisakis* may contribute to the severity. In Japan, *Anisakis* allergy accounts for 23.7% of anaphylaxis cases since people consume a high proportion of raw fish, such as in sushi; however, the accuracy of diagnosis by different doctors has become an issue.^22^ Therefore, it is important to raise awareness of *Anisakis* allergy in regions of common occurrence such as Japan, since it can contribute to anaphylaxis severity.

The proportion of drug allergies as a cause of anaphylaxis tended to increase with age, but the proportion of cases with drug allergies in the older age group was lower than that in another epidemiological study.^4^ The reason for this is that many cases of drug allergy in the elderly occur in hospital, and patients with in-hospital allergies are diagnosed and treated in various departments, and some do not visit specialist outpatient clinics. The statistical data in this study were collected from patients who visited the respiratory medicine and allergology outpatient clinic of our university hospital, so there were few patients with in-hospital drug allergies, and many cases of anaphylaxis occurring outside the hospital setting. A similar trend has also been observed in research reports from other facilities that investigated the epidemiology of anaphylaxis in adults.^23^

This study has several limitations. First, it was a single-center retrospective analysis, which may limit the generalizability of our findings—particularly given that all participants were of Japanese ethnicity, without consideration of racial or cultural diversity. Nonetheless, several of our findings align with those reported in European registry studies,^4^ suggesting that our results do not markedly diverge from broader global trends. Second, we could not collect detailed quantitative data on the number of cigarettes smoked (e.g., pack-years), exact alcohol consumption, asthma severity, or other potential cofactors/risk factors (e.g., COPD, premenstrual syndrome, NSAID use, psychological stress, fever/acute infection, and mastocytosis). This omission precluded a fully comprehensive analysis of their relationship with anaphylaxis severity. To address these gaps, we have initiated a prospective multicenter study, which includes a more rigorous assessment of smoking and alcohol use, a separate severity scoring system and wider patient demographics and results are awaited.

## Conclusions

In conclusion, age, smoking history, asthma, and alcohol consumption were significant risk factors contributing to heightened anaphylaxis severity among adults in Japan. Conversely, a history of allergies other than asthma and psychiatric disorders may not play a contributory role. Ongoing prospective observation of cases exhibiting factors associated with anaphylaxis severity (as highlighted in this study) and investigations into the course and prognosis of these cases will facilitate a more precise exploration of risk factors. Additionally, we plan to investigate the mechanisms through which the risk factors identified in this study contribute to disease exacerbation in animal models of anaphylaxis. The ultimate objective of allergy research is to decrease the incidence of fatalities resulting from anaphylaxis. The insights derived from this study will assist in identifying patients who require more intensive care, further contributing to the overarching goal of improving patient outcomes.

## Abbreviations

ACE-I/ARBs, angiotensin-converting enzyme inhibitors/angiotensin II receptor blockers; CI, confidence interval; COPD, chronic obstructive pulmonary disease; CVD, cardiovascular disease; IgE, immunoglobulin E; NSAIDs, non-steroidal anti-inflammatory drugs; OR, odds ratio; TSLP, thymic stromal lymphopoietin; WAO, World Allergy Organization.

## IRB statement

This study was approved by the Showa University Ethics Committee (Institutional Review Board number: M3136) in accordance with the Ethical Guidelines for Medical and Health Sciences Research Involving Human Subjects of the Ministry of Health, Labor, and Welfare on May 07, 2022. The requirement for obtaining informed consent from patients was waived by the ethics committee because no invasive procedures, interventions, or human samples were used in this retrospective study and anonymity was secured. However, we provided participants with opportunities to opt out of the study by announcing the study information on the bulletin boards on the hospital and hospital websites.

## Submission declaration

All authors have approved the submission of this manuscript. The results have not been previously published and are not being considered for publication in another journal.

## Data availability statement

The data that support the findings of this study are available from the corresponding author on reasonable request.

## Author contributions

Study Design: MN, SS, TU, YM, AT, and HS. Data collection: MN, SS, TU and YM. Data analysis: MN, SS, and AT. Statistical analyses: MN. Results interpretation: MN and SS. Manuscript drafting: MN and SS. All authors read and approved the final manuscript.

## Declaration of competing interest

The authors have no conflict of interest to declare.
